# Progressive hypoxia decouples activity and aerobic performance of skate embryos

**DOI:** 10.1093/conphys/cov067

**Published:** 2016-01-22

**Authors:** Valentina Di Santo, Anna H. Tran, Jon C. Svendsen

**Affiliations:** 1 Museum of Comparative Zoology, Harvard University, Cambridge, MA 02138, USA; 2 Department of Biology, Boston University, Boston, MA 02215, USA; 3 Interdisciplinary Centre of Marine and Environmental Research, University of Porto, Porto, Portugal

**Keywords:** Climate change, critical oxygen saturation, elasmobranch, *Leucoraja erinacea*, metabolism, performance

## Abstract

Although fish population size is strongly affected by survival during embryonic stages, our understanding of physiological responses to environmental stressors is based primarily on studies of post-hatch fishes. Embryonic responses to acute exposure to changes in abiotic conditions, including increase in hypoxia, could be particularly important in species exhibiting long developmental time, as embryos are unable to select a different environment behaviourally. Given that oxygen is key to metabolic processes in fishes and aquatic hypoxia is becoming more severe and frequent worldwide, organisms are expected to reduce their aerobic performance. Here, we examined the metabolic and behavioural responses of embryos of a benthic elasmobranch fish, the little skate (*Leucoraja erinacea*), to acute progressive hypoxia, by measuring oxygen consumption and movement (tail-beat) rates inside the egg case. Oxygen consumption rates were not significantly affected by ambient oxygen levels until reaching 45% air saturation (critical oxygen saturation, *S*_crit_). Below *S*_crit_, oxygen consumption rates declined rapidly, revealing an oxygen conformity response. Surprisingly, we observed a decoupling of aerobic performance and activity, as tail-beat rates increased, rather than matching the declining metabolic rates, at air saturation levels of 55% and below. These results suggest a significantly divergent response at the physiological and behavioural levels. While skate embryos depressed their metabolic rates in response to progressive hypoxia, they increased water circulation inside the egg case, presumably to restore normoxic conditions, until activity ceased abruptly around 9.8% air saturation.

## Abstract (precis)

We evaluated the usefulness of long-term plant carbon economy of a xerophyte shrub as a tool in conservation. Reserves manipulation through defoliation decreased reproduction in the long-term but not growth. Root and shoot reserves can be used as indicators of how much biomass can be harvested without threatening future reproduction

## Introduction

Many marine ecosystems are expected to experience physicochemical changes associated with increasing anthropogenic greenhouse gas emissions (Intergovernmental Panel on Climate Change, 2013). Such changes extend beyond global warming and include reduction in pH (i.e. ocean acidification), alterations in circulation patterns, increased stratification and more frequent and prolonged hypoxic (i.e. low-oxygen) events (Perry *et al.*, 2005; Cheung *et al.*, 2013; Intergovernmental Panel on Climate Change, 2013). In fact, increased stratification and reduced mixing are expected to result in significant declines in dissolved oxygen, especially in semi-enclosed bodies of water, such as bays (Bakun *et al.*, 2015). As oxygen is key to sustain every aerobic activity, it is important to understand whether and how marine organisms might tune their metabolic performance to cope with more frequent and intense hypoxic events. This is particularly crucial for benthic organisms inhabiting near-coastal waters as these are expected to be much more affected by hypoxia than highly mobile, pelagic species that are likely to possess the locomotory capacity required to sustain migrations to refugia (Lauder and Di Santo, 2015). At the same time, perhaps reflecting adaptations to typically low-oxygen environments, benthic organisms are generally thought to be able to tolerate moderate-to-severe levels of hypoxia (Seibel, 2011; Heinrich *et al.*, 2014). Despite this fact, populations of several benthic species have already declined during the last decades, and low oxygen levels are often implicated in the impairment of vital activities, including foraging and reproduction (Schurmann and Steffensen, 1997; Behrens and Steffensen, 2007; Svendsen *et al.*, 2012).

When faced with low oxygen levels, fishes can respond either by decreasing oxygen consumption rates (i.e. oxyconformity response) or by maintaining the same oxygen consumption rates despite the decrease in available dissolved oxygen in the water (i.e. oxyregulatory response). One simple and commonly adopted metric to identify species-specific sensitivity and tolerance to low oxygen levels is to quantify the oxygen saturation in the ambient water that triggers the switch from oxyregulation to oxyconformity response (Speers-Roesch *et al.*, 2012). The progressive drop in metabolic rates seen during severe hypoxia is caused by oxygen deficiency and is associated with the transition to anaerobic metabolism (Pörtner, 2010). The oxygen saturation (or pressure) point at which quiescent aquatic ectotherms are unable to maintain the normoxic metabolic rate is termed ‘critical oxygen saturation’ (*S*_crit_). The capacity of a fish to extract oxygen from the ambient water and survive acute hypoxic events is reflected in its specific *S*_crit_, with high *S*_crit_ indicating a high sensitivity (and low tolerance) to low oxygen levels (Speers-Roesch *et al.*, 2012). Correspondingly, *S*_crit_ represents an important ecological tipping point to understand the resilience of populations to declining levels of oxygen (Mueller *et al.*, 2011).

Knowing at which point species conform to oxygen levels in the water is not only important to understand the capacity of fishes to respond to challenges of hypoxia (Urbina *et al.*, 2012), but also to assess assumptions for disparate metabolic theories in ecology (Kearney and White, 2012). Although it is becoming clear that the vast majority of adult and juvenile fishes are capable of coping physiologically with a moderate decrease in ambient oxygen (Ultsch *et al.*, 1981; Virani and Rees, 2000; Perry *et al.*, 2009; Kearney and White 2012; Urbina *et al.*, 2012; Svendsen *et al.*, 2014), the metabolic responses of embryos remain uncertain. This could be particularly important in fishes that have a long developmental time, as they are most likely to experience hypoxic events at some point during embryogenesis. Most juvenile and adult fishes can respond to acute hypoxia by using a series of tactics, including moving to a different area, increasing ventilation rates, reducing swimming speed, performing aquatic surface respiration and fuelling metabolic processes through anaerobic pathways (Pollock *et al.* 2007; Chapman and McKenzie 2009; Poulsen *et al.*, 2011; Svendsen *et al.*, 2012; Genz *et al.*, 2013). In contrast, embryonic fishes have a reduced array of choices when facing hypoxia. Given that embryos are spatially constrained inside the egg case and cannot select a different habitat until they hatch, they are most likely to respond to acute hypoxia by increasing ventilation rate or by depressing aerobic metabolism. Additionally, it might be adaptively advantageous to hatch precociously in order to locate a better habitat if the ontogenetic development is sufficiently advanced to ensure survival outside of the egg case (Petranka *et al.*, 1982; Czerkies *et al.*, 2001).

The little skate, *Leucoraja erinacea* (Mitchill, 1825), is a benthic oviparous elasmobranch that inhabits near-coastal water (down to 90 m depth) in the northwestern Atlantic, from the Gulf of Maine to Cape Hatteras (Bigelow *et al.*, 1953). According to the International Union for Conservation of Nature (IUCN), *L. erinacea* is near threatened because of declining population, and the species is likely to become overfished by commercial fisheries in the near future. Female *L. erinacea* is reproductive year-round and produces large embryos individually surrounded by jelly inside leathery protective egg cases deposited on sandy or muddy flats (Leonard *et al.*, 1999; Di Santo, 2015). The egg case has horns with tendrils that secure it to the substrate (Bigelow *et al.*, 1953). After elasmobranch embryos develop external gill filaments, the jelly plug inside the egg case dissolves and the embryo starts to whip its tail to facilitate the passage of oxygenated clean water inside and through the case (see https://youtu.be/eziPYb1INA0; Thomason *et al.*, 1996; Tullis and Peterson, 2000; Hoff, 2009; Di Santo, 2015). This tail-beat activity, vital for survival and development of the embryo, is associated with an increase in metabolism (Leonard *et al.*, 1999) and it is strongly affected by climate-related stressors, including temperature and acidification (Di Santo, 2015). The combination of long developmental time (5–12 months; Di Santo, 2015) and the low likelihood to relocate to more favourable habitats given the high philopatry observed in the species (although no study to date has quantified the capacity of this fish to sustain migrations; Lauder and Di Santo, 2015) could have crucial consequences for the resilience of *L. erinacea* in possible near-future hypoxic scenarios.

In the present study, we quantified the oxygen consumption and tail-beat rates of embryonic *L. erinacea* during normoxia and progressive hypoxia. We hypothesized that skate embryos experiencing hypoxia might respond by using physiological and behavioural tactics in the following order: (i) increasing tail-beat rates as an attempt to circulate water and restore normoxia inside the egg case; (ii) switching from oxygen regulation to oxygen conformity; and (iii) hatching prematurely to escape the egg case. To quantify these responses, we used intermittent flow respirometry to measure oxygen consumption while the embryos were continuously whipping their tail in normoxic conditions and continued the measurements during incremental reductions in oxygen.

## Materials and methods

### Experimental animals

Newly laid (<1-week-old) little skate embryos (*n* = 6) were obtained from the Marine Biological Laboratory, Woods Hole, MA, USA. Embryos were transported to Boston University using a well-aerated, thermally controlled container. Once at Boston University, they were held each in independently filtered tanks, at constant salinity (33 ppt, obtained by mixing Instant Ocean^®^ and deionized water), temperature (15°C), pH (8.1) and photoperiod (14 h light–10 h dark) in a cold environmental room (Harris Environmental Systems, Inc., Andover, MA, USA). Throughout the development, skate embryos were supplied with normoxic water (∼95% air saturation). Skate embryos were used for experimentation when the egg case was open and the yolk sac diameter was ≤1 mm to ensure that the embryos were close to hatching (∼6 months at 15°C; Di Santo, 2015). The yolk size was estimated visually by shining a red light through the semi-opaque egg case and measuring the yolk diameter with digital callipers. This late developmental stage was selected to examine the possibility that hypoxia could trigger hatching and allow the skate to search for a more favourable habitat.

### Hypoxia experimental set-up

To decrease oxygen content in water, we employed a steady oxygen depletion system based on nitrogen displacement (Bennett and Beitinger, 1995; Svendsen *et al.*, 2014). Briefly, nitrogen gas from a cylinder tank was bubbled into a covered holding tank to remove oxygen. The flow of nitrogen into the tank was controlled with a Fisher Scientific nitrogen regulator, a one-way valve and a gas bubbler. While maintaining constant temperature (15°C), salinity (33 ppt) and pH (8.1), dissolved oxygen was decreased by 5% in a stepwise fashion to examine the responses to different oxygen levels (*n* = 18) between 95 and 10% air saturation.

### Oxygen consumption and tail-whip frequency

Embryos were placed and oriented ventrally in the experimental tank 4 h prior to data collection to ensure that measurements were taken in unstressed individuals (Leonard *et al.*, 1999; Di Santo, 2015). Measurements of the oxygen consumption rate ($M_{O_{2}}$; in milligrams of O_2_ per kilogram per hour) while the embryos were continuously beating their tail in the egg case [here defined as active metabolic rate (AMR) in accordance with previous studies; see Leonard *et al.*, 1999; Di Santo, 2015] were conducted using a custom-made Plexiglas^®^ intermittent flow respirometer connected with a flush and a recirculating pump similar to previous studies (Svendsen *et al.*, 2012; Tirsgaard *et al.*, 2015). The total volume of the empty respirometer was 0.701 l but was adjusted for the water displacement caused by each skate egg case and the oxygen probe. The temperature was maintained constant (15 ± 0.1°C) throughout the experiment by submerging the respirometer chamber into a water bath connected to a digitally controlled Aqua Logic chiller.

For each measurement of AMR, oxygen saturation was adjusted to match the desired level in the chamber while the flushing and the circulation pumps were actuated. Embryos experienced each saturation condition for 30 min. Following this period, the flushing pump was turned off and oxygen decline in the respirometer was measured for 20 min. Oxygen concentration was measured using a YSI oxygen meter (model 550A) calibrated daily. Measurements of AMR continued until skate embryos ceased whipping their tail for at least 1 min. Activity rates (tail beats per minute) were recorded for 1 min at 5 min intervals at each tested oxygen level by shining a red light through the semi-opaque egg case. After each trial, skates were returned to normoxic conditions and gently extracted from the egg case using blunt forceps inserted into the anterior slit. Once extracted, skates were weighed to record wet mass to the nearest 0.01 g. Oxygen consumption rates were calculated based on the slope of a linear regression of the decline in oxygen concentration inside the chamber during the 20 min measurement phase. Measurements were adjusted by mass using the coefficient (0.67) previously tested and applied for this species and other elasmobranchs (Di Santo and Bennett, 2011a; Di Santo, 2015) using the following equation:

$$\begin{array}{ll} M_{O_{2}}(\mathrm{mg}O_{2}\;kg^{-1}\;h^{-1}) & =\mathrm{Slope}\;O_{2}\;\mathrm{change}(\mathrm{mg}\;O_{2}\;l^{-1}\;h^{-1})\\ & \times \;\mathrm{Volume}(l)\times \mathrm{Mas}s^{-0.67}\;(\mathrm{kg}) \end{array}$$

Blank respirometry trials were conducted with the empty egg cases for 1 h, but background respiration was never detected (*n* = 6).

### Statistical analysis

Analyses of oxygen consumption and tail-beat rates in relationship to O_2_ saturation levels were conducted using a repeated-measures ANOVA, followed by Dunnett’s test (control: air saturation of 95%) to estimate the oxygen levels that triggered a change in metabolic and activity rates. All statistical analyses were based on α = 0.05 and were undertaken in JMP Pro, version 11 (SAS).

## Results

**Figure 1:**
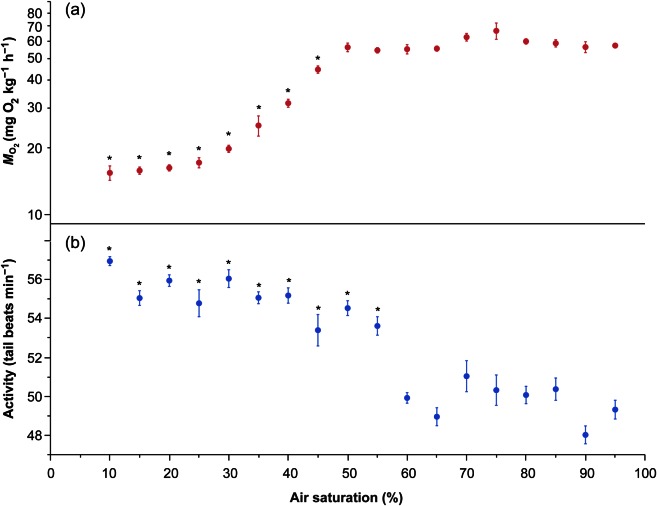
Near-hatch embryonic little skate (*Leucoraja erinacea*; *n* = 6) oxygen consumption ($M_{O_{2}}$; **a**) and activity (**b**), assessed as tail-beat rate (means ± SEM) as a function of decreasing oxygen in the environment (air saturation, expressed as a percentage). Asterisks show statistically significant difference in mean $M_{O_{2}}$ and activity rates from normoxic levels, 95% air saturation (repeated-measures ANOVA, followed by Dunnett’s test; α = 0.05).

Hypoxic conditions (at and below 45% air saturation; *S*_crit_) significantly reduced AMR in little skate embryos, indicating an oxygen conformity response (repeated-measures ANOVA, *F*_85,17_ = 102.45; *P* < 0.000001; Fig. 1a). However, a stepwise decrease in oxygen did not significantly affect AMR between 95 and 50% air saturation (Dunnett’s test, α = 0.05), indicating an oxygen regulating response. Skate embryos maintained similar tail-beat rates across oxygen saturations down to 55%, after which the rate increased (repeated-measures ANOVA, *F*_85,17_ = 30.81; *P* < 0.000001; Dunnett’s test, α = 0.05; Fig. 1b). Surprisingly, there was no gradual decrease in tail-beat frequency as a function of progressive hypoxia. Instead, we observed an abrupt complete cessation of all movement at 9.8 ± 0.05% air saturation (i.e. tail-beat collapse). All skates recovered and survived the procedure, but hypoxia did not trigger hatching, despite the fact that aerobic metabolism was depressed and activity ceased completely at the end of the experiment.

## Discussion

Over recent decades, hypoxic events have increased in frequency, severity and geographical extent and it is therefore crucial to know the response of fish performance at different levels of hypoxia (Herbert *et al*., 2011). A reduction in AMR at oxygen levels below *S*_crit_ forces fishes to use anaerobic pathways and has detrimental effects on activity and resilience of species (Schurmann and Steffensen, 1997; Claireaux *et al.*, 2000; Chabot and Claireaux, 2008). The present study indicates that embryos of *L. erinacea* are highly sensitive to hypoxia, because they decrease oxygen consumption at air saturation levels of 45%, while activity in the egg case increases, possibly to enhance ventilation and exchange of water with the surrounding environment. Combining metabolic and behavioural measurements, our results show that embryos of *L. erinacea* maintained constant metabolic rates during activity down to 45% (*S*_crit_), revealing an oxygen regulating response to changing oxygen levels. However, at oxygen saturations below *S*_crit_, metabolism declined abruptly and significantly with increasing levels of hypoxia, suggesting an oxygen conformity response and, possibly, a progressive reliance on anaerobic metabolism. Interestingly, activity was more sensitive to decreasing oxygen than metabolic rates, because tail-beat rates increased at higher levels of oxygen in the water (55%). Thus, this behavioural switch occurred well above *S*_crit_, indicating that the first response to oxygen decline in the environment is behavioural rather than metabolic. Furthermore, activity did not decrease before completely collapsing at the end of the trial. This result is in contrast to previous studies, because complete cessation of hyperventilation in hypoxic conditions is uncommon in fishes (Perry *et al.*, 2009).

It is widely assumed that when the aerobic scope for activity is limited by the reduction in oxygen in the ambient water, fishes will attempt to reduce energetic expenditure by decreasing movement (Chabot and Claireaux, 2008). Although this assumption has been substantiated by studies on relatively sluggish species of the genera *Gadus* (Schurmann and Steffensen, 1997), *Carassius* (Nilsson *et al.*, 1993) and *Solea* (Dalla Via *et al.*, 1998), it was not confirmed in more active species of the genera *Tunnus* (Bushnell and Brill, 1991), *Oncorhynchus* (Poulsen *et al.*, 2011) and *Cynoscion* (Brady *et al.*, 2009). Based on these few studies, there seems to be an apparent correlation between ‘physiotype’ (e.g. active vs. inactive fishes) and activity regulation (Burggren and Randall, 1978; Metcalfe and Butler, 1984; Cook *et al.*, 2011; Di Santo and Bennett, 2011b). In fact, active fishes might upregulate their locomotory performance to escape hypoxic environments, whereas more sluggish physiotypes might downregulate activity in order to reduce energy costs during a temporary oxygen crisis, and thus decrease the chance of incurring lethal metabolic deficits. Although it might be expected that benthic fishes, such as skates, would respond to a decrease in oxygen levels by reducing both activity and metabolic rates, embryos seem to exploit a different tactic, i.e. they significantly increase tail-beat rate down to a point where activities can no longer be sustained. During severe hypoxia, activity was elevated by ∼10% from normoxic conditions, but metabolic rates were depressed up to ∼70%. This suggests a decoupling of activity and aerobic metabolism, as embryos were possibly becoming progressively more reliant on anaerobic pathways, a strategy that could not be sustained for a prolonged period of time (Pörtner and Farrell, 2008).

Studies on the effect of hypoxia on fish embryos are scarce but they suggest that both acute and chronic exposure to hypoxia may result in a decrease in survival and growth and an increase in developmental time and malformations (Podrabsky *et al.*, 2001; Shang and Wu, 2004; Breitburg *et al.*, 2009). A few studies on the effect of hypoxia on elasmobranchs suggest that some species in this group may even be sensitive to a brief acute exposure to low oxygen levels. For example, the shovelnose ray (*Aptychotrema rostrata*) cannot tolerate oxygen tensions of 2 kPa for 30 min (Speers-Roesch, 2012). On the contrary, the epaulette shark (*Hemiscyllium ocellatum*) exhibits an extraordinary tolerance to hypoxia, which may be given by the capacity to minimize changes in fluid acidosis and cardiac function (Speers-Roesch, 2012). In the present study, all *L. erinacea* embryos survived the acute exposure to hypoxia but did not hatch spontaneously and needed to be extracted from the egg cases to be weighed and measured. In response to hypoxia, some fishes may hatch prematurely, whereas others may in fact delay hatching (Hassell *et al.*, 2008). For instance, precocious hatching has been observed in fishes of the genus *Coregonus* (Czerkies *et al.*, 2001), *Oncorhynchus* (Ciuhandu *et al.*, 2005) and *Acanthopagrus* (Hassell *et al.*, 2008). However, these same studies observed delayed hatching as well. It is plausible that a similar phenomenon occurred in *L. erinacea*, where most fish did not respond to low ambient oxygen by hatching (or escaping the egg case) even though anecdotal evidence suggests that early hatching could occur (V.D.S., personal observation). In fact, during preliminary studies in which skate embryos were maintained in a closed respirometer and allowed to consume oxygen without flushing, they exited the egg case at ∼2 mg O_2_ l^−1^ (at 15°C; V.D.S., personal observation). However, other abiotic factors, such as CO_2_ and ammonia, were not controlled, and skates might have hatched in response to these stressors rather than low oxygen levels.

*Leucoraja erinacea* embryos are enclosed in a capsule throughout the relatively long development (up to 1 year), and it is therefore critical for the survival of this species that embryos possess the capacity to surmount hypoxic events in their habitat using various mechanisms. If hypoxic events continue to increase in frequency and to expand geographically in the northwestern Atlantic (Fulweiler *et al.*, 2012), these could potentially affect fitness and resilience of populations of *L. erinacea*. As reproduction in the fish is not timed with seasons but occurs year-round (Palm *et al.*, 2011) and the species shows strong philopatry (Frisk and Miller, 2006, 2009), female *L. erinacea* are not expected to modify reproductive timing or spawning location (Di Santo, 2015; Lauder and Di Santo, 2015). Moreover, warming could further exacerbate the effect of hypoxia because metabolic processes require more oxygen at higher temperatures (Perry *et al.*, 2005). However, in the present study, temperature and other abiotic factors (pH and salinity) were kept constant, so the shift between oxyregulation and oxyconformity should be attributed to oxygen levels alone.

It is clear that for large embryos, stuck in the egg case for several months, tail-beat activity is the only means by which water is circulated and moved though the egg case and, ultimately, normoxia is maintained. As other tactics, such as precocious hatching, are only observed occasionally, prolonged aquatic hypoxia could severely impair development of embryonic oviparous elasmobranchs, with a possible increase in pre-hatching mortality. Nevertheless, an approach whereby physiological and behavioural responses to climatic stressors are combined with spatial and temporal data on abiotic stressors (i.e. hypoxia, warming and acidification) could help to identify vulnerable areas and potential refugia, thus improving conservation outcomes for oviparous sharks and skates.

## Funding

This project was funded by grants from the American Fisheries Society, the American Society of Ichthyologists and Herpetologists, the American Elasmobranch Society, Flying Sharks, The Oceanário de Lisboa, and the Portuguese Association for the Study and Conservation of Elasmobranchs to V.D.S.; while conducting the experiments and writing the manuscript, V.D.S. was supported by the Warren-McLeod Research, Dana Wright and Ryan Kelley Fellowships; A.H.T. was supported by the UROP program at Boston University; and J.C.S. was supported by a grant (SFRH/BPD/89473/2012) from the Foundation for Science and Technology (FCT) in Portugal.
